# It takes a village to form embryo models

**DOI:** 10.1016/j.stemcr.2021.04.014

**Published:** 2021-05-11

**Authors:** Nicolas Rivron, Jianping Fu

**Affiliations:** Institute of Molecular Biotechnology of the Austrian Academy of Sciences, Vienna BioCenter, Vienna, Austria; Department of Mechanical Engineering, Department of Biomedical Engineering, Department of Cell & Developmental Biology University of Michigan, Ann Arbor, Ann Arbor, MI 48198, USA

## Main text

How do cells orchestrate growth and forms? New entry points into this classical question have emerged through the formation of embryo models made solely from stem cells. These models are more scalable, versatile, and accessible than mammalian embryos, opening up new avenues for investigating knowledge gaps in embryogenesis and organogenesis (see in this issue Rossant and Tam, 1031). Understanding the basic principles of proliferation, differentiation, patterning, and morphogenesis not only fulfills our curiosity but also settles ground rules for tissue engineering, regenerative medicine, and disease modeling (see in this issue Moris et al., 1021).

Embryo models are especially relevant for species whose development is poorly understood due to inaccessibility, scarcity, or ethical status. This is particularly true for mammalians, including humans, and non-model organisms, including rare species. Although embryo models were initially developed using mouse and human stem cells, this field can extend to other species, broadening the space for discoveries about the “endless forms most beautiful and most wonderful,” as described by Charles Darwin.

**Figure 1:**
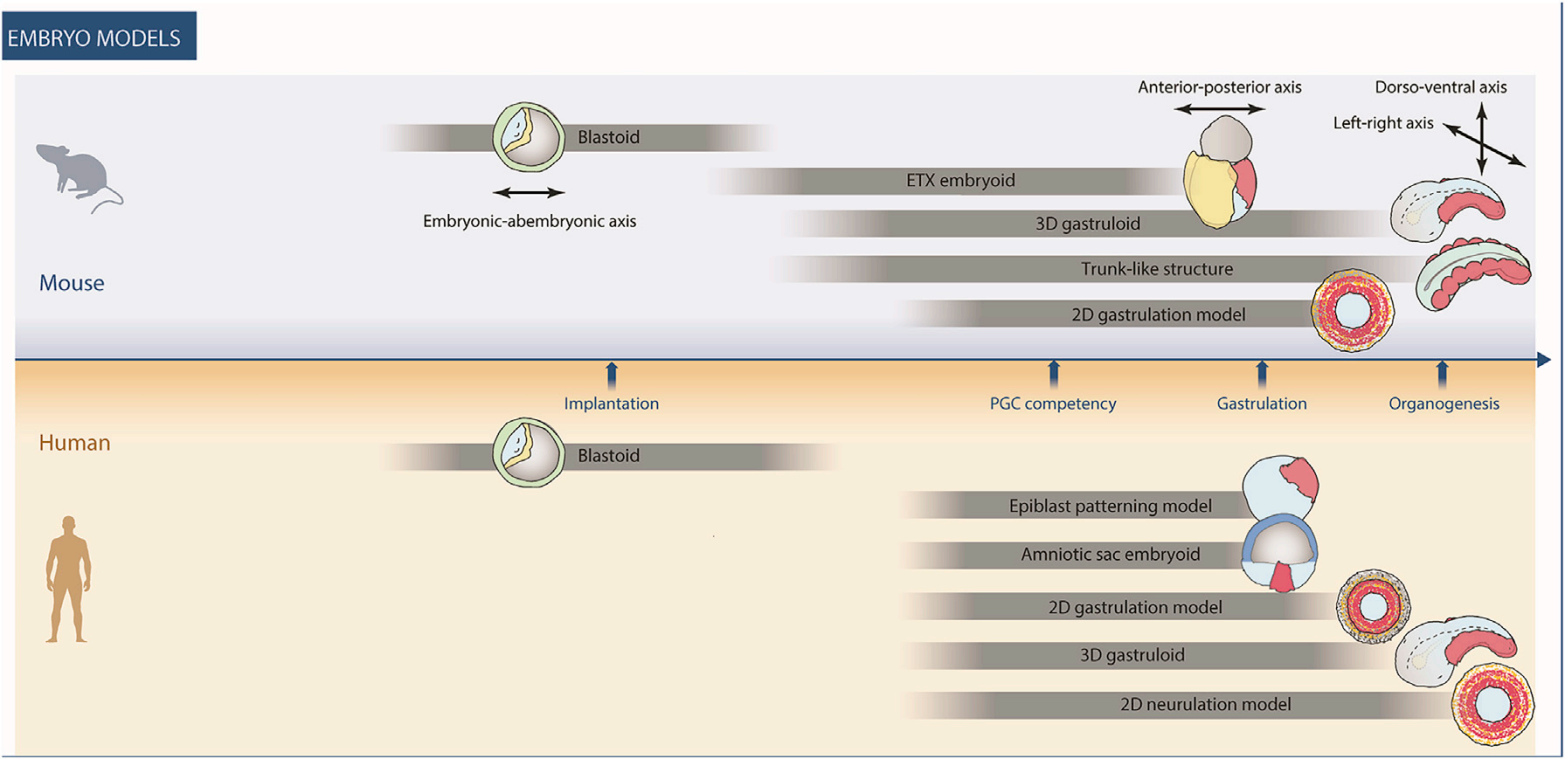
Stem cells have the intrinsic capacity to re-enact multicellular behaviors reminiscent of early embryogenesis When adequately guided, stem cells can efficiently form embryo models with different levels of completeness and reflecting different stages. These models not only allow an understanding of the fundamental principles of proliferation, differentiation, patterning, and morphogenesis, but also settle common ground rules for tissue engineering, regenerative medicine, and disease modeling (see Snapshot in this issue, 1142).

The origin of embryo models can be traced back to embryoid bodies, aggregates of pluripotent stem cells undergoing lineage specification, which proved very useful for validating their differentiation potential. However, embryoid bodies form rather disorganized tissues, which prevents developmental progression. This limitation prompted efforts from Yoshiki Sasai and others in more finely directing pluripotent stem cells to recapitulate aspects of organogenesis. Continuing in this vein, embryo models are generated by combining pluripotent stem cells, sometimes along with extraembryonic stem cells, in precisely controlled numbers, geometries, and physicochemical environments mimicking developmental cues. This triggers cells to re-enact aspects of embryogenesis reflecting the formation of the pre-implantation blastocyst and post-implantation gastrula, all the way to early organogenesis including the development of the nervous system and the heart (Figure 1).

**Self-organization**. Embryogenesis has historically been viewed through the lens of pre-patterning, whether by local deposition of maternal RNA or by mechanochemical inductions often originating from extraembryonic tissues. This knowledge of embryonic-extraembryonic interactions has been repurposed to form embryo models and tissue decoupling of has refined our understanding of inductions. It has also highlighted a certain level of tissue autonomy and arguably underappreciated processes of self-organization. Such a theoretical framework has accompanied progress in embryology, as exemplified by the reaction-diffusion models of Alan Turing and Hans Meinhardt. Lately, testing these theories in embryo models has revealed the dynamic propagation of signaling activities underlying patterning processes that are likely to contribute to gastrulation (see in this issue Liu et al., 1065). How inductions and self-organization interact to guide development will most likely refine the principles of embryogenesis (see in this issue Morales et al., 1039).

**Engineering development**. Admixing stage-matched embryonic and extraembryonic stem cells can recreate dynamic microenvironments and behaviors occurring within embryos. These microenvironments can also be engineered *in vitro*. Neighboring tissues within embryo models can be replaced by the inductive signals they normally produce, by using microfluidics, bioprinting, or micropatterned surfaces. These tools also improve the controllability, reproducibility, and scalability of embryo models (see in this issue Gupta et al., 1104). In addition to external signals, synthetic biology, making use of engineered gene circuits or rewiring natural ones, provides novel tools for controlling, perturbing, and recording cell behaviors from within. The rapid advance of synthetic biology has already demonstrated applications in non-developmental minimal systems and in organisms. In the future, it could be utilized in embryo models to inform on the properties of genes and proteins networks and to tune their formation (see in this issue Ho et al., 1051).

**Achieving high-fidelity**. An immediate challenge is to construct high-fidelity embryo models that are optimized and benchmarked to natural embryos. This is crucial to ensure that the knowledge obtained from these models is scientifically and clinically relevant. Such an effort starts with a detailed understanding of the developing embryo, which became feasible thanks to recent advances in single-cell genomic and *in toto* imaging. Due to the limited accessibility of human embryos, investigations into non-human primate development (see in this issue Nakamura et al., 1093) and non-primate bilaminar disc embryos, whose developments show similarities with human embryos, will provide important reference points (see in this issue Alberio et al., 1078). Capturing stem cell states that more closely resemble the *in vivo* counterparts is also crucial. Indeed, current stem cell cultures are often heterogeneous, reflecting multiple developmental stages, and states that are not found *in vivo* (e.g. epigenetic and metabolic). In this issue, Posfai et al. (1117) propose the establishment of quality standards to evaluate to which extent the initial stem cells and final models resemble the *in vivo* counterparts. All together, high-fidelity stem cells and optimal culture environments ensure a timely unrolling of developmental processes. A too-long or uncoordinated process often points at suboptimal starting stem cells or culture conditions for the embryo model.

**Biomedical applications**. Embryo models can inform about how to improve human health in a variety of ways.

1. It is estimated that at least 40% of pregnancies end before 20 weeks of gestation, with 70% of those failing around implantation. An improved understanding of early embryogenesis could inform on therapeutic strategies to improve infertility treatments.
2. Only about 20% of IVF procedures result in a birth. Understanding early embryogenesis could instruct on ways to optimize the implantation of IVF embryos while minimizing cellular abnormalities. This could reduce the number of IVF procedures while ensuring the health of children conceived by IVF.
3. The ability for women to control pregnancy is critical to sustainable, global development and to the improvement of gender equality. Knowledge about early embryogenesis could lead to the development of effective contraceptive strategies with fewer side effects.
4. Subtle abnormalities in the first weeks of pregnancy, such as those caused by the use of alcohol or medications, can have long-term consequences. For example, genetic mosaicism or abnormal placental development can limit embryo growth, affect birth weight, and increase the propensity for chronic diseases during adult life. Embryo models could aid in identifying the underlying mechanisms and assessing the effects of diets or drugs.
5. Forming organs in an environment resembling that of the developing embryo could lead to the formation of organ rudiments that more closely mimic complex, mature, and functional organs. As such, embryo models could help to generate interconnected tissues and organs for drug screens or transplantation or help to understand and promote regeneration. (See in this issue Moris et al., 1021).

**Ethical framework**. Finally, it is important to mention that embryo models are currently rudimentary and different from embryos. They do not arise from the fusion of gametes and can only mimic a short developmental time window of typically a few days. As such, they are not considered equivalent to intact embryos under most legislation. Nevertheless, embryo model research necessitates a framework for ethical oversight, as proposed in the ISSCR ethical guidelines. Also, the terms *embryo model* or *embryoid* probably best reflect the current state and envisioned applications of these entities. Considering the proportionality (balancing the benefits and harms) and subsidiarity (pursuing goals using the morally least problematic means) of embryology, embryo models represent an ethical alternative complementary to the use of embryos (see in this issue Rossant and Tam, 1031 and Matthews et al., 1014).

It will take a village to form high-fidelity embryo models. Gathering a community comprising stem cell biologists, embryologists, synthetic biologists, computational biologists, physicists, engineers, and ethicists will be key to establish firm grounds. We believe that careful embryo model development will lead to a better understanding of embryogenesis, to the identification of therapeutic targets and preclinical modeling for precision medicine, and to guidance for tissue engineering and regenerative medicine.

**Figure 2:**
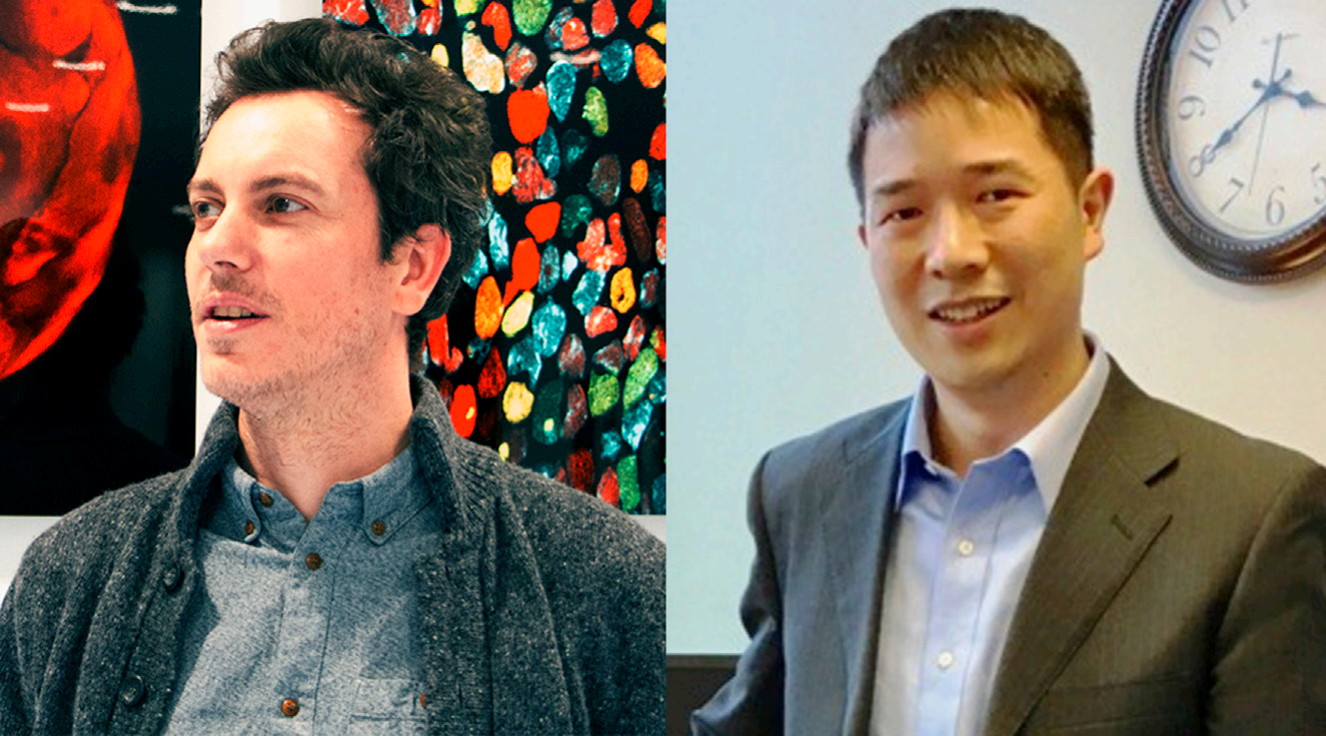
Nicolas Rivron (left) and Jianping Fu (right) are the guest editors for this special issue of *Stem Cell Reports*

This special issue on embryo models is developed in parallel with an ISSCR digital series on the same topic, which brings together many of the authors from this issue and additional experts in the field for a discussion of these exciting themes. We as guest editors (Figure 2) would like to thank the authors for their contributions to this issue and *Stem Cell Reports* for featuring this important area of research.

